# Role of Vitamin D and Vitamin D Receptor in Oral Lichen Planus: A Systematic Review

**DOI:** 10.4314/ejhs.v30i4.17

**Published:** 2020-07-01

**Authors:** Paria Motahari, Fatemeh Pournaghi Azar, Arefeh Rasi

**Affiliations:** 1 Assistant Professor, Department of Oral Medicine, Faculty of Dentistry, Tabriz University of Medical Sciences, Tabriz, Iran; 2 Assistant Professor, Department of Restorative Dentistry, Faculty of Dentistry, Tabriz University of Medical Sciences, Tabriz, Iran; Dentist, Department of Oral Medicine, Faculty of Dentistry, Tabriz University of Medical Sciences, Tabriz, Iran

**Keywords:** Oral lichen planus, Vitamin D, Vitamin D receptor

## Abstract

**Background:**

Oral lichen planus (OLP) is known to be a chronic inflammatory disease associated with various other systemic disorders. Studies have shown that vitamin D deficiency can be involved in the pathogenesis of lichen planus. The aim of this study was to investigate the role of vitamin D and vitamin D receptor in OLP

**Methods:**

In this review study, all English and Persian articles were searched by relevant keywords from the Google scholar, PubMed, science direct, Cochrane, Scopus and Sid databases until January 2020.

**Results:**

From the 16 articles obtained after reviewing the abstracts, finally 14 appropriate articles were included in this study.

**Conclusion:**

According to the results of the studies, vitamin D deficiency may be associated with an increased risk of OLP lesions.

## Introduction

Lichenoid reactions represent a family of lesions with different etiologies with a common clinical and histologic appearance. Oral lichenoid reactions include the following disorders: Oral lichen planus (OLP), Lichenoid contact reactions, Lichenoid drug eruptions and Lichenoid reactions of graft-versus-host disease (GVHD) (1). OLP is known to be a chronic inflammatory disease associated with various other systemic disorders (2). It often involves middle-aged patients and is more common in women than in men (3). OLP is commonly seen as symmetrical and bilateral lesions with multifocal involvement in the oral mucosa (4). The etiology of the disease is not yet well understood. In different systemic conditions and various autoimmune diseases and under the use of drugs and exposed to a number of substances and infections, lesions similar to lichen planus occur, but the exact relationship between all these factors and the cause of the lesion has not been determined yet (5). An important issue with this disease is the possibility of malignant changes. This has been the subject of debate for many years. Although extensive research has been done in this area, it is still doubtful whether the lesion is benign or susceptible to malignancy. OLP has various clinical appearances, which can occur in different places of the mouth, can be uncomfortable or accompanied by burning and pain and may show periods of recurrence and recover. This variation in clinical appearance can make diagnosis difficult (6).

Vitamin D is a fat-soluble vitamin that has two internal and external sources (7,8). Although until two decades ago, there was no conception of the relationship between vitamin D and the immune system; increasing studies in recent years suggest that this vitamin's role in the immune system is widespread. Vitamin D receptors (VDR) are abundantly present in T lymphocytes and macrophages and most commonly in immature thymic immune cells and mature TCD8 + lymphocytes (9–11). Different forms of Vitamin D can play a role in suppressing of autoimmune diseases in animal models. In this regard, the results of studies indicated that 1 and 25 -Hydroxyvitamin D3 can prevent or suppress the manifestation of experimental autoimmune encephalomyelitis, rheumatoid arthritis, systemic lupus erythematous, diabetes, and inflammatory bowel disease (12,13). The mechanism of these suppressive effects against autoimmune diseases has also been suggested that vitamin D causes production of interleukin-4 (IL-4) and Transforming growth factor-beta (TGFB-1) which suppresses T-cell inflammatory activities. Vitamin D deficiency in the immune system reduces T helper 2 (Th2) levels and other cells involved in the inflammatory pathway such as Th1 and Th17 (14).

In recent years, numerous studies have been conducted on the role of vitamin D in the prevention of OLP. Studies have shown that vitamin D inhibits the production of interferon gamma (IFN-γ) and IL-1β in the epithelium and its deficiency may be involved in the pathogenesis of OLP (15–18). Epidemiologic studies showed that conflicting results regarding the serum levels of vitamin D and the role of this vitamin and its receptors in the development of OLP. In some of these studies, no significant difference was found between serum vitamin D levels and healthy controls, and in other studies, vitamin D deficiency was found in individuals with OLP compared to healthy controls (15–18). In this study, we aim to systematically investigate the role of vitamin D and its receptor in OLP. Identification of this role in OLP might be useful for development of new preventive and therapeutic methods in management of patients with OLP.

## Methods

### Search strategy

This systematic review was conducted based on the Preferred Reporting Items for Systematic Reviews and Meta-Analyses (PRISMA) statement for reporting systematic reviews (19).

In this review study, all published English and Persian articles with the keywords of "oral lichen planus" and "vitamin D receptor" or "VDR" and "vitamin D" and their Persian equivalents from Google scholar, PubMed, Science Direct, Cochrane, Scopus and Sid databases were searched until January 2020. In the initial phase, the titles and abstracts of the articles were reviewed by two independent individuals based on the inclusion and exclusion criteria. Disagreements were resolved with the third author’s discussion. Next, the full text of the selected articles was reviewed. The quality of the studies was evaluated by the Newcastle-Ottawa scale method (20). The data of selected articles were extracted using data extraction form. This form includes the author's name/year of publication/sample size and the results of the studies.

### Inclusion criteria

The inclusion criteria are the studies that 1) have evaluated serum levels of vitamin D in patients with active phase of OLP who have recently been diagnosed with the disease and did not previously received medication for OLP; 2) have examined the effect of vitamin D on OLP; 3) have investigated VDR gene polymorphism in patients with OLP and 4) have investigated the mechanisms of creating OLP due to Vitamin D or VDR Deficiency.

### Exclusion criteria

The review and case reports articles and studies that have investigated the role of vitamin D or its receptor in patients with cutaneous lichen planus were excluded.

### Statistical analyses

The analyses were done by Review Manager 5.3 (Rev- Man 5.3, the Cochrane Collaboration, Oxford, UK) with a random-effects model using mean difference (MD) and 95% confidence intervals (CIs). The heterogeneity percentage between the studies was evaluated by the Cochrane Q test and I2 statistic; if I2 > 50%, there was a heterogeneity. P value =0.05 was considered statistically significant.

## Results

**Figure 1:**
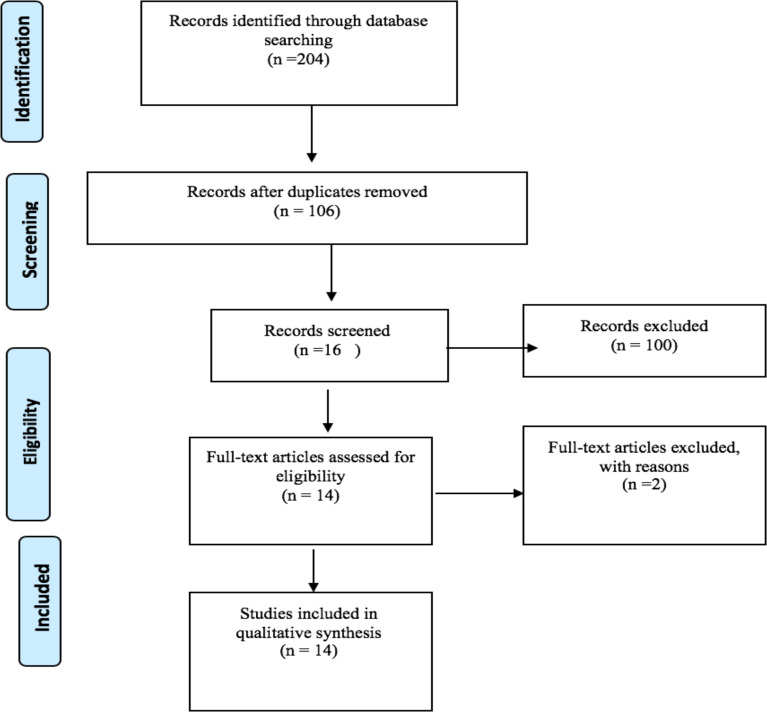
The flowchart of searching strategy based on PRISMA guidelines

Initial searches of 106 articles were extracted. From the 16 articles obtained after reviewing the abstracts, finally 14 appropriate articles were included in this study based on the entry and exit criteria shown in Figure 1. Of the 14 articles selected, 4 studies examined serum levels of vitamin D in affected individuals (17,18,21,22), 2 studies studied the effect of vitamin D supplementation on OLP symptoms (23,24), in 2 studies, the association of VDR gene polymorphisms and OLP risk have been investigated (25,26) and 6 studies have investigated the mechanisms of OLP formation due to vitamin D deficiency (27–32) (Table 1).

**Table 1:** summary of the data extracted from included studies in this review

Studies groups	Authors	Results
A: Studies that have evaluated serum levels of vitamin D in patient with OLP	Gupta et al/2017 (21)	102 patients with OLP and 102 healthy subjects were included in study. Vitamin D in the cases of OLP and control was 20.40 ng / ml and 32.67 ng / ml, respectively. This difference was statistically significant
	Muzaffar et al/2017 (18)	20 patients with OLP and 20 healthy subjects were included in study. Statistically significant difference was seen in vitamin D level of serum between healthy subjects and patients with OLP (P = 0.05)
	Bahramiyan et al/2018 (17)	18 patients with OLP and 18 healthy subjects were included in study. The mean vitamin D level in serum of patients with OLP was 30.38 ± 20.38 ng / ml and in healthy subjects 36.45 ± 15.33 ng / ml, there was no statistically significant difference. (P = 0.34)
	Seif et al/2018 (22)	30 patients with OLP and 66 healthy subjects were included in study. Serum vitamin D levels are decreased in a high percentage of patients with OLP.
B: Studies that have evaluated the effect of vitamin D supplementation on OLP symptoms	Razi et al/2018 (23)	100 women in premenopausal period with vitamin D serum level below 30 ng/ml divided into 2 groups: group A which received routine treatment and group B which received routine treatment + vitamin D supplement. Their results showed Subjects in group B shows improvement in clinical appearance of lesion between week 1 and week 4.
	Gupta et al/2019 (24)	106 patients with OLP divided to 3 groups: group 1 received topical steroid treatment with psychological counseling, group 2 received topical steroids with vitamin D supplementation and group 3 received topical steroids with vitamin D supplementation and psychiatric consultation. Their results showed a statistically significant improvement in symptoms (mentally and objectively) in patients receiving vitamin D supplementation.
C: Studies that have investigated the association of VDR and OLP gene polymorphisms	Kujundzic et al/2016 (25)	VDRFokI rs2228570 gene polymorphism significantly increased OLP risk in allelic, genotypic and dominant models, which was not significant for VDR EcoRV rs4516035, VDRApaI rs7975232 and VDRTaqI rs731236 polymorphisms.
	Shen et al/2020 (26)	VDR rs2239185 polymorphism in TT and recessive genotypes and VDR rs7975232 gene polymorphism in CC and recessive genotypes significantly increased OLP risk.
D: Studies showing the pathways of OLP lesions due to vitamin D or VDR deficiency	Du et al/2017 (27)	Vitamin D and its associated VDR have an anti-inflammatory role in OLP through regulation of the nuclear factor-γB (NF-γB) pathway. VDR expression is reduced in Th1-mediated diseases.
	Zhao et al/2018 (28)	Lipopolysaccharides (LPS) decrease VDR through the tumor necrosis factor alpha (TNFα) - miR-346 pathway. Vitamin D / VDR signaling suppresses LPS-induced p53-upregulated modulator of apoptosis (PUMA) via NF-κB pathway blockade, thereby reducing apoptosis of epithelial cells
	Zhao et al/2019 (29)	Vitamin D / VDR signaling suppresses LPS-induced hypoxia-inducible factor-1α (HIF-1α) via the NF-κB pathway block, thereby reducing the production of IFN γ and IL-1β.
	Ge et al/2019 (30)	Vitamin D / VDR signaling inhibits miR-802 expression by regulating the NF-κB pathway, thereby reducing apoptosis of epithelial cells. miR-802 Increases Bcl-2 Expression (Bcl-2) and Induces Apoptosis of Epithelial Cells
	Ge et al/2020 (31)	MicroRNA-27a / b significantly decreased in serum, saliva and OLP tissue samples. They showed that there are sites for VDR binding in the promoter region of the MicroRNA-27a / b gene and that Vitamin D / VDR signaling induces MicroRNA-27a / b in OLP.
	Du et al/2020 (32)	MicroRNA-26a / b significantly decreased in serum, saliva and OLP tissue samples. They showed that there are sites for VDR binding in the promoter region of the MicroRNA-26a / b gene and that Vitamin D / VDR signaling induces MicroRNA-26a /b in OLP. MicroRNA-26a / b has a protective role in OLP through inhibition of apoptosis and reduction of pro-inflammatory cytokines in epithelial cells.

### Serum vitamin D levels

**Figure 2:**
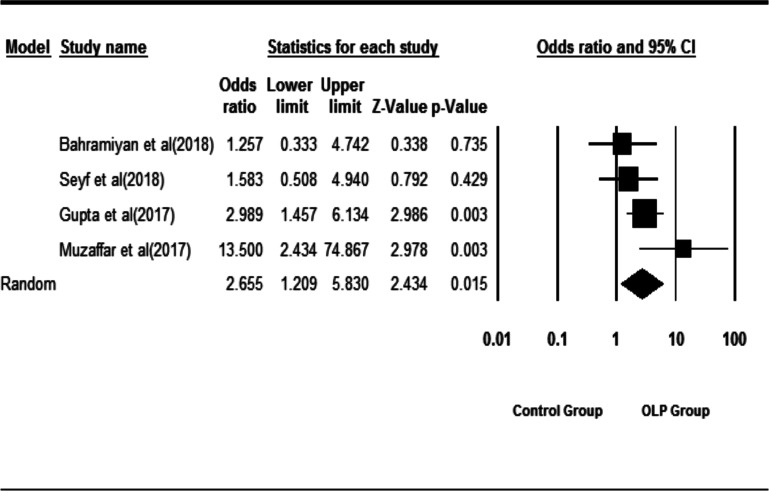
Forest diagrams from the meta-analysis

Figure 2 shows the forest diagrams of the meta-analysis. Four studies were included in the meta-analysis. In the OLP and control groups, 206 and 170 patients were studied, respectively. There were 166 patients with OLP and 104 controls with vitamin D less than 30 ng/ml. Heterogeneity between studies was not significant (Q = 5.57, df = 3, I2 = 46.19, p-value = 0.13). According to the results of the meta-analysis, the odds of vitamin D levels in patients with OLP being lower than 30 ng/ml were 2.65 times higher than those in the control group (OR = 2.65.95% CI = 1.20 -5.83, p-value = 0.015).

### The effect of vitamin D supplementation on OLP symptoms

 Studies that have evaluated the effect of vitamin D supplementation on OLP showed statistically significant improvement in symptoms of OLP patients receiving vitamin D supplementation.

### Association of VDR and OLP Gene Polymorphisms

As a ligand-dependent transcription factor, vitamin D receptors encoded by the VDR gene (chromosomal locus 14-12q12) play important roles in regulating the role of vitamin D (33). Significant differences in allelic distribution and genotype of VDR FokI polymorphism (rs2228570) between OLP patients and control group indicated the possible importance of VDR FokI polymorphism in susceptibility to OLP. The single nucleotide polymorphisms are rs2239185 and rs7975232 in the intron region of the VDR gene (34). The GTEX database has shown that the amount of VDR expression in the blood is actually affected by genetic changes. Thus, polymorphisms in VDR genes may affect the expression of blood VDR and decrease the binding of vitamin D to VDR in blood and oral keratinocytes and prevent activation of the VD/VDR pathway (29). Such anti-inflammatory effects are inhibited and eventually lead to lichen planus. In two studies by Kujundzic et al (25) and Shen et al (26) on the polymorphism of the rs7975232 VDR gene, different results were presented. It is noteworthy that the different results may be due to different ethnic populations and sample sizes.

### Pathways of creating OLP due to Vitamin D or VDR Deficiency

**Figure 3:**
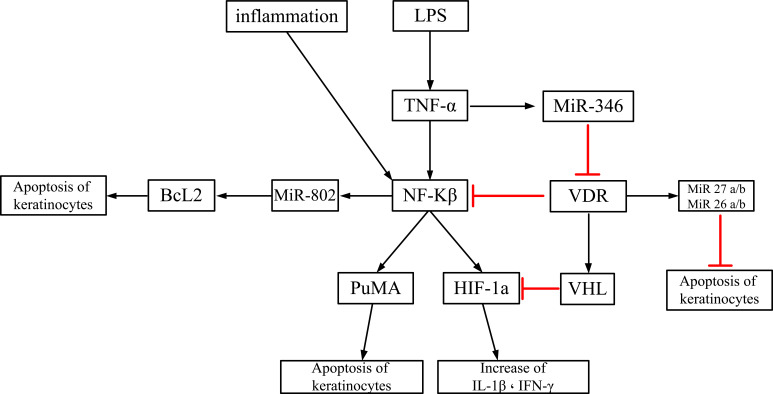
Schematic illustration of mediatory effect of vitamin D/VDR signaling on prevention of OLP

Schematic illustration of mediatory effect of vitamin D/VDR signaling on prevention of OLP is shown in Figure 3.

## Discussion

In the present study, the role of vitamin D and VDR in OLP was investigated. The results of this review study showed that vitamin D deficiency was present in a higher percentage of patients with OLP, and that the use of vitamin D supplements caused a significant improvement in these patients. Various properties of vitamin D have been investigated for its effects in different types of diseases. In addition, the effects of vitamin D can be affected by the presence of single nucleotide polymorphisms in vitamin D-related genes, such as the VDR and cytochrome P450 genes that are involved in vitamin D metabolism.

Because OLP is considered a potential precancerous lesion, single nucleotide polymorphisms (SNPs) in VDR or vitamin D pathway genes may also play important roles in oral cancer. Hormones 1 and 25 -Hydroxyvitamin D3 induce VDR receptor expression in epithelial cells and act as a mucosal barrier. The VDR receptor protects against invasion of microbes into the underlying connective tissue, and in the case of the oral lichen planus, epithelial destruction means apoptosis or planned cell death (15). Vitamin D deficiency results in impaired T cell proliferation and may therefore lead to the development of OLP. The conclusion from Figure 3 is that inflammation and bacterial lipopolysaccharides activate pro-inflammatory cytokines and produce factors that induce apoptosis of keratinocytes and the formation of OLP lesions. Chinese researchers have found that vitamin D/VDR signaling inhibits the NFκB pathway and also increases the production of MicroRNA-26, 27 a/b. As a result, apoptosis of epithelial cells and the possibility of OLP lesions are reduced (27–32).

In developed or developing countries, vitamin D insufficiency or deficiency among the general population has been the focus of much research. This may be due to a variety of reasons, including increased use of sunscreen, increased indoor activity, and more skin coverage due to different cultural beliefs and religious practices or protection against skin cancer. The conclusion to be drawn from this study is that vitamin D deficiency may be associated with an increased risk of developing OLP lesions. It is hoped that this study will raise our awareness of the role of vitamin D and its receptor in OLP lesions and open new avenues of treatment and prevention.
